# A Pilot Study of Echinocandin Combination with Trimethoprim/Sulfamethoxazole and Clindamycin for the Treatment of AIDS Patients with Pneumocystis Pneumonia

**DOI:** 10.1155/2019/8105075

**Published:** 2019-12-01

**Authors:** Mengyan Wang, Guanjing Lang, Ying Chen, Caiqin Hu, Yongzheng Guo, Ran Tao, Xiaotian Dong, Biao Zhu

**Affiliations:** ^1^The Department of Infectious Diseases, State Key Laboratory for Diagnosis and Treatment of Infectious Diseases, Collaborative Innovation Center for Diagnosis and Treatment of Infectious Diseases, National Clinical Research Center for Infectious Diseases, The First Affiliated Hospital, College of Medicine, Zhejiang University, Hangzhou, China; ^2^Xixi Hospital of Hangzhou, Hangzhou 310023, China

## Abstract

**Background and Objectives:**

Pneumocystis pneumonia (PCP) is a common opportunistic infection in acquired immune deficiency syndrome (AIDS) patients that continues to result in a high mortality rate. To develop a better treatment strategy and improve PCP prognosis, a cohort study was conducted to evaluate the therapeutic potential of echinocandin treatment for AIDS patients with PCP (AIDS-PCP).

**Methods:**

The AIDS-PCP patients were analyzed in our retrospective cohort study that were hospitalized in The First Affiliated Hospital of Zhejiang University during 2013–2018. The antifungal effects of echinocandins were evaluated in two subgroups that were classified by oxygenation as a proxy for the disease state: PaO_2_/FiO_2_ > 200 mmHg and PaO_2_/FiO_2_ ≤ 200 mmHg. Intergroup comparisons and survival curves were used to evaluate the effectiveness of the two AIDS-PCP treatment regimens.

**Results:**

During the follow-up, 182 AIDS-PCP patients were diagnosed and analyzed in the study. After excluding 55 patients with other superinfections and five patients that were treated with HAART, the remaining 122 patients were enrolled in the study. The group treated with echinocandins combined with trimethoprim-sulfamethoxazole (TMP-SMZ) and clindamycin exhibited a lower mortality rate (9.62%, 5/52) than did the group with TMP-SMZ and clindamycin treatment (20%, 14/70). For AIDS-PCP patients in the PaO_2_/FiO_2_ > 200 mmHg subgroup, treatment with echinocandins combined with TMP-SMZ and clindamycin significantly reduced their mortality rate (4.44% (2/45) vs. 18.18% (10/55), *P* = 0.035).

**Conclusion:**

The results of this study indicate that treatment with echinocandins in combination with the standard TMP-SMZ and clindamycin regimen can improve the prognosis and reduce the mortality rate in patients with mild to moderate AIDS-PCP disease.

## 1. Introduction

Human pneumocystis pneumonia (PCP) is caused by *Pneumocystis jirovecii*. In patients with immunodeficiencies or that are undergoing immunosuppression, latent cells will rapidly multiply and destroy alveolar cells, thereby causing interstitial pneumonia [1]. PCP is a life-threatening opportunistic infection [2] that is the most common cause of AIDS-related deaths (20.3–47.7% of all deaths) [3, 4]. Although AIDS-related mortality within a year of initiating ART was 7.44 per 100 person-years which has declined owing to the routine use of highly active antiretroviral therapies (HAART), the risk of early death is still as high as 31.8% [4, 5]. TMP-SMX is the currently used first-line drug for the prevention and treatment of PCP. Alternative drugs include pentamidine, dapsone plus trimethoprim, primaquine plus clindamycin, and atovaquone. In addition, glucocorticosteroid adjuvant therapy can help mechanical ventilation and reduce the mortality rate within patients exhibiting moderate to severe PCP [6, 7]. Despite advances in the prevention and management of PCP, the serious side effects and drug resistance associated with existing treatment regimens require consideration [8]. Increasing numbers of studies have indicated that mutations in dihydropteroate synthase genes may be associated with the emergence of TMP-SMX resistant strains [9]. Alternative candidate drugs including echinocandins like caspofungin have been investigated thoroughly for therapeutic potential in treating PCP. Caspofungin is an antifungal agent that acts on spore cysts by inhibiting the synthesis of *β*-(1,3)-D-glucan [10]. Lee et al., Li et al., and Lu et al. observed that echinocandins in combination with TMP-SMX treatment can improve the prognosis of PCP patients and decrease the associated mortality rate [11–13]. However, there has been little investigation of these effects in AIDS-PCP patients. Consequently, the current study was designed to evaluate the therapeutic potential of a combined echinocandins/TMP-SMX treatment for AIDS-PCP patients.

## 2. Materials and Methods

### 2.1. Study Population

A total of 182 AIDS-PCP patients were investigated that were hospitalized in The First Affiliated Hospital of Zhejiang University between January 2013 and June 2018. Inclusion criteria include (1) confirmed AIDS diagnosis, (2) age ≥ 18 years, (3) naive adults with a first episode of AIDS-PCP, and (4) PCP diagnosis. Diagnosis for PCP includes (1) insidious or subacute onset, dry cough, shortness of breath, and increased postactivity and may have fever, progressive dyspnea, and purpura; (2) chest computed tomography suggests increased lung texture, coarse, strip-like shadows, or scattered small patchy shadows or diffuse reticular nodular interstitial infiltration or frosted glassy shadows; (3) hypoxemia; (4) elevated level of blood lactate dehydrogenase; and (5) TMP-SMX treatment responding well. Exclusion criteria included: (1) patients that received HAART at the onset of the disease; (2) the presence of other immunocompromised diseases including tumors, congenital diseases, and prior chemotherapy treatment; (3) immune reconstitution inflammatory syndrome (IRIS)patients; (4) the presence of severe allergies or allergies to sulfa drugs; (5) the presence of severe heart, brain, liver, kidney, or other important organ diseases; and (6) the presence of severe blood and endocrine system diseases or past medical history of these diseases. All patients provided informed consent, and this study was approved by the Ethics Committee of The First Affiliated Hospital, College of Medicine, Zhejiang University (reference number 2017471).

**Figure 1:**
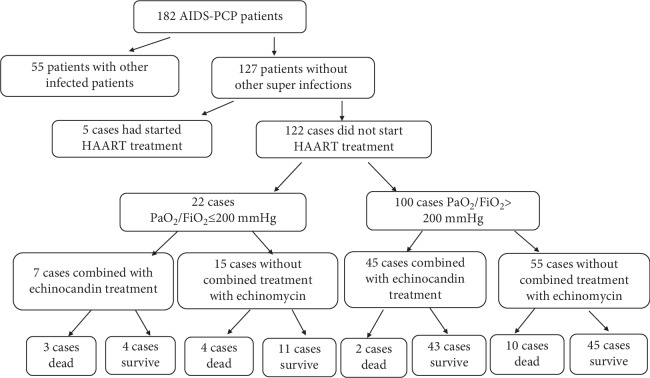
Flow chart indicating the process underlying patient selection and group classification.

To specifically investigate the therapeutic effects of echinocandins in AIDS-PCP patients, 55 patients with other superinfections and five who had begun antiretroviral therapy were excluded. Of the remaining 122 patients, 52 were provided echinocandins/TMP-SMX/clindamycin treatment and 70 patients were treated with TMP-SMX and clindamycin treatment. And all these patients accepted the treatment of glucocorticoid. Patients were divided into two subgroups according to the patient's oxygenation index: PaO_2_/FiO_2_ > 200 mmHg (*n* = 100) and PaO_2_/FiO_2_ ≤ 200 mmHg (*n* = 22). A schematic indicating the work flow of patient inclusion and classification is shown in Figure 1.

### 2.2. Clinical Information

Clinical data were collected for each subject including demographic characteristics, treatment, superinfections, clinical outcomes, and the results of laboratory tests within 12 h of admission. The tests comprised blood tests, biochemical assays, D-dimer, ferritin, CRP, LDH, HBDH, CD4 cell counts, and blood gas analyses.

### 2.3. Statistical Methods

Nonparametric Mann–Whitney *U* tests or Student's *t*-tests were used for data analysis when data were nonnormal or normal, respectively. Kolmogorov-Smirnov tests were used for normal distribution. Chi-squared tests, continuity corrections, or Fisher's exact tests were used to test statistical significances for categorical data. Binary logistic regressions were used in multivariate analyses to predict mortality. Kaplan-Meier and log-rank tests were used to analyze and compare survival rates. All statistical analyses were performed using SPSS version 19 (SPSS, Armonk, New York, USA) using a statistical significance threshold of *P* < 0.05.

## 3. Results

### 3.1. Demographic Characteristics and Mortality

A total of 182 AIDS-PCP patients were admitted to The First Affiliated Hospital of Zhejiang University between January 2013 and June 2018. Of the remaining 122 patients, 52 were provided echinocandins/TMP-SMX/clindamycin treatment and 70 patients were treated with TMP-SMX and clindamycin treatment. Clinical characteristics and baseline demographics of the 122 patients between combined echinocandins and noncombined echinocandins are shown in Table 1.

### 3.2. Intergroup Comparisons of Echinocandins/TMP-SMX/Clindamycin and TMP-SMX/Clindamycin Treatments

**Figure 2:**
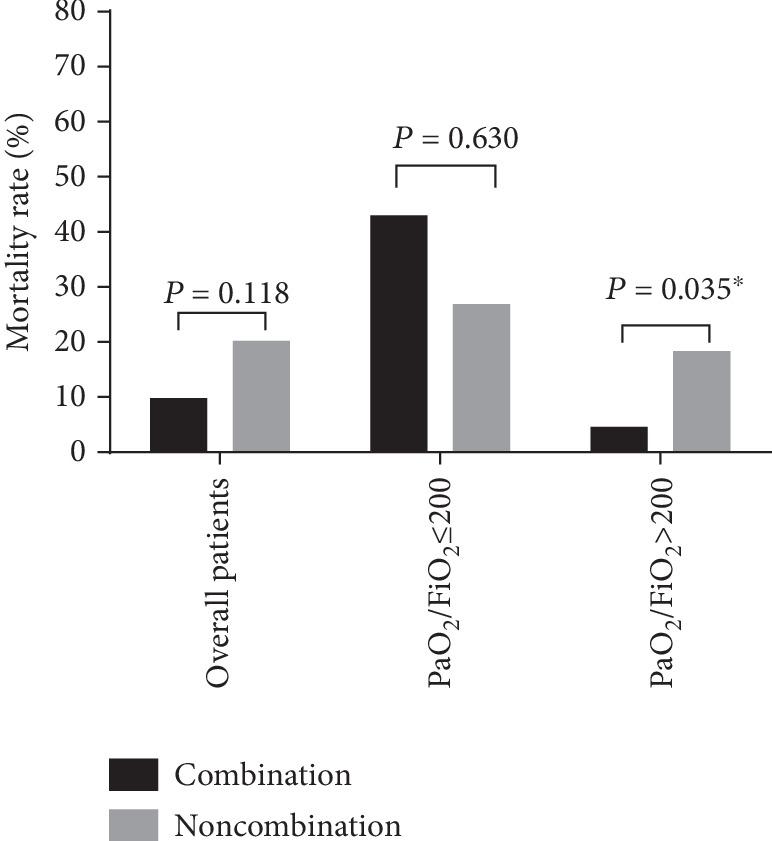
Mortality rates of patients with treatments of either combined echinocandins or noncombined echinocandins.

Among all 122 patients investigated in the study, those within the echinocandins/TMP-SMX and clindamycin treatment group exhibited a mortality rate of 9.62% (5/52), while those within the TMP-SMX and clindamycin treatment group exhibited a mortality rate of 20% (14/70). Despite the difference in mortality outcomes, the difference was not statistically significant (*P* = 0.118; Figure 2).

In the subgroup of patients with PaO_2_/FiO_2_ ≤ 200 mmHg (*n* = 22), those with echinocandins/TMP-SMX and clindamycin treatment exhibited a mortality rate of 26.67% (4/15), while the rate was higher, 42.86% (3/7), for those within the TMP-SMX and clindamycin treatment group. However, the difference between the two treatment groups was not statistically significant (*P* = 0.630).

In the subgroup of patients with PaO_2_/FiO_2_ > 200 mmHg (*n* = 100), those within the echinocandins/TMP-SMX and clindamycin treatment group exhibited a significantly lower mortality rate (4.44%, 2/45) than did those in the TMP-SMX and clindamycin group (18.18%, 10/55; *P* = 0.035).

### 3.3. Survival in Patients with Mild AIDS-PCP

**Figure 3:**
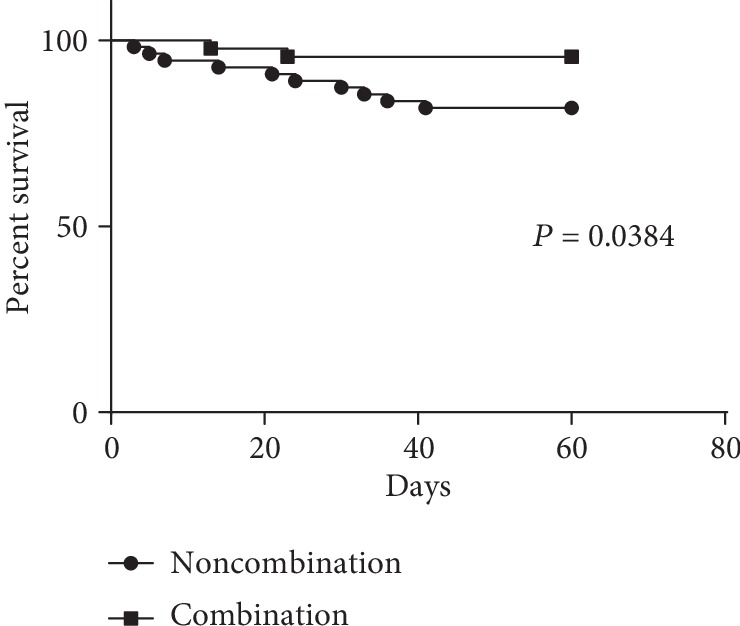
Survival in AIDS-PCP patients exhibiting mild disease states (PaO_2_/FiO_2_ > 200 mmHg) after treatment. Two subgroups were considered based on either combined echinocandin treatment or noncombined echinocandins.

Survival rates were analyzed and compared between patients in the echinocandins/TMP-SMX/clindamycin and TMP-SMX/clindamycin treatment groups based on clinical outcomes within two months for patients with PaO_2_/FiO_2_ > 200 mmHg. Patients within the TMP-SMX/clindamycin treatment group exhibited a significantly lower survival rate than did those in the echinocandins/TMP-SMX/clindamycin group (*P* = 0.0384; Figure 3).

## 4. Discussion

Most AIDS-PCP patients are in advanced stages and have high risks of contracting various opportunistic infections [14]. To adequately investigate the role of echinocandins and TMP-SMX treatments and reduce confounding factors caused by other superinfections and the corresponding treatments, we excluded patients with superinfections from the study cohort. These infections included tuberculosis, *Cryptococcus*, CMV, Epstein-Barr virus, hepatitis B virus, and syphilis. The glucocorticoid can improve clinical outcome in AIDS patients with PCP. All patients investigated in this study accepted treatment of glucocorticoid for reducing factors of the impacting clinical outcome.

Echinocandins inhibit the synthesis of *β*-(1,3)-D-glucan and block the formation of pneumocystis cysts, although they are less effective against trophozoite forms [15]. These observations suggest that echinocandins can reduce pathogen reservoirs. In addition, TMP-SMX inhibits trophozoite forms of Pneumocystis by interfering with their metabolism of folate. Therefore, the combination of echinocandins and TMP-SMX can inhibit the entire life cycle of Pneumocystis parasites [16]. TMP-SMX acts slowly, requiring five to eight days to achieve stable therapeutic effects [17], while echinocandins act rapidly. Consequently, the combination of TMP-SMX and echinocandins can exhibit synergistic effects in the treatment of PCP patients. Importantly, echinocandins induce less adverse events in PCP patients because they do not inhibit the CYP system or induce CYP3A4 drug metabolism [18].

The addition of echinocandins to a standard TMP-SMX regimen reduced the mortality rate among all 122 enrolled patients of this study (9.62% vs. 20%), although the difference was not statistically significant. Following this observation, patients were divided into two subgroups, PaO_2_/FiO_2_ > 200 mmHg (indicating mild disease) and PaO_2_/FiO_2_ ≤ 200 mmHg (indicating severe disease). The early application of a combined echinocandins/TMP-SMX treatment for mild AIDS-PCP patients could significantly reduce the mortality rate compared to TMP-SMX treatment alone (4.44% vs. 18.18%, *P* < 0.05). These findings are consistent with some case reports suggesting that echinocandins could improve the prognosis of patients with PCP [11–13]. In addition, the feasibility of combined echinocandins/TMP-SMX treatment for PCP was demonstrated in studies of PCP animal models and *in vitro* experiments [19–21]. A previous caspofungin salvage treatment trial indicated that caspofungin could improve the prognosis and reduce the mortality rate of AIDS-PCP patients [22]. However, there were two major limitations of this study: the lack of a control group and the small sample size (*n* = 12). However, the results of the present study confirm the observations from the trial, while using a larger sample size (*n* = 122) and an experimental design that featured a control group.

In our study, the subgroup of patients with PaO2/FiO2 ≤ 200 mmHg (*n* = 22) and those with echinocandins/TMP-SMX and clindamycin treatment exhibited a mortality rate of 42.86% (3/7), while the rate was higher, 26.67% (4/15), for those within the TMP-SMX and clindamycin treatment group. This results reported here indicate that adding echinocandins to the standard regimen could not reduce the mortality rate for critically ill patients in advanced stages. However, the sample size of patients with PaO2/FiO2 ≤ 200 mmHg was small. Lung damage resulting from PCP is very rapid, and deaths often result from a rapid increase in proinflammatory cytokines [23]. Echinocandins can inhibit the inflammatory response induced by *β*-glucan by inhibiting *β*-glucan synthesis, thereby alleviating the symptoms of PCP [24]. Consequently, early application of a combined echinocandins/TMP-SMX treatment for PCP patients with mild to moderate disease states could inhibit the inflammatory response and cytokine production, thereby reducing their mortality rate.

Our study had some limitations: this study did not perform bronchoscopic examination to make a molecular test for PCP diagnosis. However, most patients were severe. Because of hypoxia and dyspnea symptoms, it was difficult to carry out bronchoscopic examination. And we had adopted a unified clinical diagnostic standard. Additionally, limited sample size, single-center analysis, and retrospective studies may cause some bias.

## 5. Conclusions

The results of this study demonstrate that early application of combined echinocandins/TMP-SMX treatment for AIDS-PCP patients can improve patient prognosis, increase their survival rate, and decrease their mortality rate.

## Figures and Tables

**Table 1:** Clinical and demographic characteristics of AIDS-PCP patients investigated in this study, China, 2013-2018.

Demographic data	Patients, no.			*P* value
	Total patients (*n* = 122)	Combined echinocandins (*n* = 52)	Noncombined echinocandins (*n* = 70)	
Sex (M/F)	112/10	45/7	67/3	0.096
Age (y)	42.15 ± 13.90	41.43 ± 12.99	43.31 ± 15.29	0.462
Recent CD4 count (cells/*μ*L)	59.24 ± 66.86	64.64 ± 66.43	51.24 ± 67.35	0.275
Number of hospitalization days	19.93 ± 9.73	19.36 ± 10.42	20.92 ± 8.45	0.409
Treatment				
TMP-SMX (80 mg-400 mg, tid)	122	52	70	NA
Clindamycin (800 mg, q8h)	122	52	70	NA
Echinocandins (50 mg, qd)	52	52	0	NA
Glucocorticoid (40 mg, bid)	122	52	70	NA
Clinical outcome				
PaO_2_/FiO_2_> 200 mmHg/PaO_2_/FiO_2_ ≤ 200 mmHg	100/22	45/7	55/15	0.258
Survival/death	103/19	47/5	56/14	0.118

## Data Availability

The data statement support the findings of this study are available.

## References

[1] Thomas C. F., Limper A. H. (2004). Pneumocystis pneumonia. *The New England Journal of Medicine*.

[2] Oladele R. O., Otu A. A., Richardson M. D., Denning D. W. (2018). Diagnosis and management of pneumocystis pneumonia in resource-poor settings. *Journal of Health Care for the Poor and Underserved*.

[3] Wickramasekaran R. N., Jewell M. P., Sorvillo F., Kuo T. (2017). The changing trends and profile of pneumocystosis mortality in the United States, 1999‐2014. *Mycoses*.

[4] Lee S. H., Kim K. H., Lee S. G. (2013). Trends of mortality and cause of death among HIV-infected patients in Korea, 1990-2011. *Journal of Korean Medical Science*.

[5] Silverman R. A., John-Stewart G. C., Beck I. A. (2019). Predictors of mortality within the first year of initiating antiretroviral therapy in urban and rural Kenya: a prospective cohort study. *PLoS One*.

[6] Castro J. G., Morrison-Bryant M. (2010). Management of Pneumocystis Jirovecii pneumonia in HIV infected patients: current options, challenges and future directions. *HIV/AIDS - Research and Palliative Care*.

[7] Bozzette S. A., Sattler F. R., Chiu J. (1990). A controlled trial of early adjunctive treatment with corticosteroids for Pneumocystis carinii pneumonia in the acquired immunodeficiency syndrome. California Collaborative Treatment Group. *The New England Journal of Medicine*.

[8] Huang Y. S., Yang J. J., Lee N. Y. (2017). Treatment of Pneumocystis jirovecii pneumonia in HIV-infected patients: a review. *Expert Review of Anti-Infective Therapy*.

[9] Matos O., Esteves F. (2010). Epidemiology and clinical relevance of Pneumocystis jirovecii Frenkel, 1976 dihydropteroate synthase gene mutations. *Parasite*.

[10] Esteves F., Medrano F. J., de Armas Y., Wissmann G., Calderon E. J., Matos O. (2014). Pneumocystis and pneumocystosis: first meeting of experts from Latin-American and Portuguese-speaking countries - a mini-review. *Expert Review of Anti-Infective Therapy*.

[11] Lu Y. M., Lee Y. T., Chang H. C. (2017). Combination of echinocandins and trimethoprim/sulfamethoxazole for the treatment of Pneumocystis jiroveci pneumonia after heart transplantation. *Transplantation Proceedings*.

[12] Li H., Huang H., He H. (2016). Successful treatment of severe Pneumocystis pneumonia in an immunosuppressed patient using caspofungin combined with clindamycin: a case report and literature review. *BMC Pulmonary Medicine*.

[13] Lee N., Lawrence D., Patel B., Ledot S. (2017). HIV-relatedPneumocystis jiroveciipneumonia managed with caspofungin and veno-venous extracorporeal membrane oxygenation rescue therapy. *BML Case Reports*.

[14] Buchacz K., Lau B., Jing Y. (2016). Incidence of AIDS-defining opportunistic infections in a multicohort analysis of HIV-infected persons in the United States and Canada, 2000-2010. *The Journal of Infectious Diseases*.

[15] Vassallo R., Standing J. E., Limper A. H. (2000). IsolatedPneumocystis cariniiCell wall glucan provokes lower respiratory tract inflammatory responses. *Journal of Immunology*.

[16] Utili R., Durante-Mangoni E., Basilico C., Mattei A., Ragone E., Grossi P. (2007). Efficacy of caspofungin addition to trimethoprim-sulfamethoxazole treatment for severe pneumocystis pneumonia in solid organ transplant recipients. *Transplantation*.

[17] Roux A., Gonzalez F., Roux M. (2014). L'infection pulmonaire a _Pneumocystis jirovecii_ chez les patients VIH n egatifs : mise au point. *Médecine et Maladies Infectieuses*.

[18] Waters L., Nelson M. (2007). The use of caspofungin in HIV-infected individuals. *Expert Opinion on Investigational Drugs*.

[19] Powles M. A., Liberator P., Anderson J. (1998). Efficacy of MK-991 (L-743,872), a semisynthetic pneumocandin, in murine models of Pneumocystis carinii. *Antimicrobial Agents and Chemotherapy*.

[20] Cushion M. T., Collins M. S. (2011). Susceptibility of Pneumocystis to echinocandins in suspension and biofilm cultures. *Antimicrobial Agents and Chemotherapy*.

[21] Lobo M. L., Esteves F., de Sousa B. (2013). Therapeutic potential of caspofungin combined with trimethoprim-sulfamethoxazole for pneumocystis pneumonia: a pilot study in mice. *PLoS One*.

[22] Armstrong-James D., Stebbing J., John L. (2011). A trial of caspofungin salvage treatment in PCP pneumonia. *Thorax*.

[23] Kobayashi H., Worgall S., O'Connor T. P., Crystal R. G. (2007). Interaction of Pneumocystis carinii with dendritic cells and resulting host responses to P. carinii. *Journal of Immunotherapy*.

[24] Linke M. J., Ashbaugh A., Collins M. S., Lynch K., Cushion M. T. (2013). Characterization of a distinct host response profile to Pneumocystis murina asci during clearance of pneumocystis pneumonia. *Infection and Immunity*.

